# Integrating Telehealth Emergency Department Follow-up Visits into Residency Training

**DOI:** 10.7759/cureus.2433

**Published:** 2018-04-05

**Authors:** Dimitrios Papanagnou, Danica Stone, Shruti Chandra, Phillip Watts, Anna Marie Chang, Judd E Hollander

**Affiliations:** 1 Department of Emergency Medicine, Thomas Jefferson University; 2 Jeffconnect Program, Thomas Jefferson University

**Keywords:** telehealth, telemedicine, graduate medical education, training, professional development

## Abstract

Introduction

Given the rapid expansion of telehealth (TH), there is an emerging need for trained professionals who can effectively deliver TH services. As there is no formal TH training program for residents, the Department of Emergency Medicine (DEM) at Thomas Jefferson University (TJU) developed a pilot training program for senior post-graduate-year three (PGY-3) residents that exposed them to TH practices. The objective of the study was to determine the feasibility of developing a resident-led, post-Emergency-Department (ED) visit TH follow-up program as an educational opportunity to 1) address patient satisfaction; and 2) expose senior residents to TH delivery.

Methods

During a one-month block in their third-year of training, EM residents were exposed to and educated on TH delivery and utility through on-the-job, just-in-time training. Residents spent four hours per week evaluating patients previously seen in the ED within the last 5-7 days in the form of TH follow-up visits. ED patients were screened to identify which patient chief complaints and presentations were appropriate for a follow-up visit, given a specific day and time for their TH encounter, facilitated by a resident, and supervised by a faculty member trained in TH. Demographic patient and visit data were collected. Residents then completed a brief survey at the end of the rotation to capture their educational experiences and recommendations for subsequent training improvement.

Results

Over 12 months, 197 TH follow-up visits were performed by 12 residents. One hundred twenty-six patients (64%) were female. Top chief complaints included extremity pain (11.2%); abdominal pain (8.1%); upper respiratory infections (8.1%); lacerations (7.6%), and motor vehicle accidents (7.6%). The average number of days between the ED visit and the TH follow-up call was 5.1 days (IQR 3-6). 44.7% of patients were compliant with their discharge instructions and medications. On a Likert scale low (1) to high (10)], average patient helpfulness rating was 8.2 (IQR 7.8-10) and the average patient likelihood to recommend a TH follow-up visit was 8.5 (IQR 8-10). Ten residents completed the follow-up survey on the educational experience of the rotation (response rate 83%), of which seven described there is value to have a TH rotation in the curriculum. Thematic analysis of open-ended responses yielded constructive feedback for programmatic improvement.

Conclusion

The authors propose a feasible TH training opportunity integrated into EM residency training to assist them with meeting a rapidly-growing demand for TH and prepare them for diverse job opportunities.

## Introduction

Telehealth (TH) has been shown to broaden access to healthcare [1], increase efficiency while reducing costs [2-3], and enhance patient satisfaction and health outcomes. Given its rapid expansion, there is an emerging need for trained professionals who will effectively deliver TH services. More recently, TH has been used as a direct connection to the patient, rather than a physician to physician consult. The direct-to-consumer TH industry is growing rapidly with over 1 million visits in 2014 [2].

International healthcare organizations, practitioners, patients, families, policy makers, and legislators are increasingly recognizing the value added by the integrated use of telehealth. Given the high impact potential of telehealth, it is predicted that there will be an 18.5% annual growth of TH services through 2018, inevitably resulting in spiking demand for TH training and coordination [4]. The 2020 Health Report states that the introduction of new TH and telemonitoring programs in healthcare will require a new subset of skills that will change the roles and responsibilities of health providers [5].

To date, there are few academic programs that offer TH training especially for post-graduate resident trainees [6]. The University of Alaska Southeast (in partnership with Alaska Federal Health Care Access Network) is one of the few institutions in the country that offers a TH certificate program (for continuing education, CE, contact hours). The University of Arizona, University of California-Davis Health System, and Waldo County General Hospital (Maine) also run similar programs. Other non-accredited, short-term training and educational resources exist, most notably through the Cal Telehealth Resource Center (also for CE credit) and the American Telehealth Association (ATA). There are no sizeable general TH programs in the northeast United States [6].

Given telehealth’s growing market and trending demands in the United States and abroad, it is expected there will be significant implications and expectations for the emergency medicine (EM) residency graduate. It was in response to this accumulating evidence that the Department of Emergency Medicine (DEM) at Thomas Jefferson University (TJU) created and successfully integrated a TH training program into its residency program in an effort of preparing its graduates for telehealth’s rapidly-growing demand. A major focus was placed on developing senior residents’ clinical and non-clinical skills that are essential to TH delivery, such as the TH physical exam, virtual doctor-patient communication, and troubleshooting technological challenges.

The goal of this paper is to describe the first 12 months of the program, including the resident and patient experience of a novel TH follow-up encounter subsequent to the traditional ED visit. Specifically, the authors aimed to determine if such a training program could be integrated into an EM graduate medical education program to describe what patient complaints EM residents would typically encounter through a TH medium, and to survey residents on the educational value of TH training.

## Materials and methods

Our curriculum and quality improvement project was completed at TJU, an academic medical center located in Philadelphia, Pennsylvania that has an enterprise-wide telehealth program (JeffConnect) including 24/7/365 on-demand access to an emergency physician, a 38-hospital neurostroke network providing partner affiliate hospitals’ emergency physicians with telehealth access to neurovascular experts, and scheduled visit programs in 18 clinical specialties. Over 10,000 telehealth calls are completed annually.

Targeted learners

The TJU sponsors a 3-year (post-graduate year, PGY, 1 through 3), EM residency training program, with 36 total residents (12 residents per year). The residency program is accredited by the Accreditation Council for Graduate Medical Education (ACGME). During the one-month Emergency Medicine Administration and Teaching (EMAT) block of the last year of training, PGY-3 residents were immersed in a multifaceted TH experience which exposed, educated, and trained senior residents on the delivery and utility of telehealth in patient care.

With regards to the goals of the rotation, residents were expected to develop both the clinical and non-clinical skills that are essential to telehealth care delivery, such as the TH physical exam, virtual doctor-patient communication, and troubleshooting technological challenges. At the beginning of the rotation, residents were given an introductory orientation providing them with an overview of the TH program. Residents were provided with resources on telehealth principles. These resources included internally-developed modules that introduced telehealth, its applications, the telehealth physical exam, troubleshooting, and regulatory considerations; institution-specific clinical pathways; and video examples of successful TH encounters.

Residents spent four hours per week performing follow-up visits on patients seen in the ED within the past five to seven days. ED patients appropriately identified for a follow-up visit were given a specific day and time for their virtual telehealth encounter, which was facilitated by the PGY-3 resident, and supervised by a faculty member credentialed in telehealth delivery.

Patient recruitment

ED patients suitable for a telehealth follow-up visit were identified by a faculty member, resident, and/or research coordinator. Patients were enlisted for a TH follow-up visit only if the treating physician agreed that a timely follow-up visit would be helpful for the patient and would assist in the management of his/her chief complaint. Chief complaints were low-acuity, and included upper respiratory infections, wound care concerns, lacerations, cellulitis, headaches, or minor trauma, as examples. If an ED patient was amenable to participate in a virtual visit, a research coordinator then scheduled the patient for the virtual appointment prior to his/her discharge. All patients were also contacted 30 days after their TH visit to assess whether their respective issues were resolved or further care was necessary, and to gauge their experience with the TH encounter.

Assessment and supervision

Each resident was expected to complete 16 hours of directly supervised TH delivery over the four-week period (4 hours per week x four weeks). Evaluations of residents were performed by the supervising faculty member. All supervising faculty members hold a faculty appointment in the DEM are trained in TH and provide ongoing TH coverage within the enterprise-wide JeffConnect program. Formative feedback was provided to each resident during and at the completion of each TH encounter.

The telehealth visit

Prior to the TH encounter, residents reviewed the patient’s ED note from their original visit to the hospital. All scheduled visits were conducted via video using a HIPAA-compliant, secure, JeffConnect platform (www.jeffconnect.org), which can be conducted via the web, smart phone, or tablet device. At the time of scheduling, a coupon code was created, which made the follow-up visit free to the patient. When logging-on to the platform, patients entered structured clinical information, including any medications and allergies. Once this information was successfully entered, patients clicked a button that immediately connected them to the physician. The average wait time for this process is one to three minutes. Physicians and patients were then able to view one other, similar to a video call on Skype or FaceTime. Documentation was captured in the electronic medical record, identifying the encounter as a TH visit, and linking it to the original ED visit as an addendum to the patient's medical record.

Data collection

During the telehealth encounter, residents followed a standardized approach to collecting patient data, which included medication reconciliation, assessment of whether ED discharge follow-up instructions were followed, and an assessment of how the patient was doing since his/her discharge from the ED. Any issues or questions that patients had were also addressed. Data collected from each encounter included the number of patient visits to the ED, chief complaint, days between initial visit and telehealth visit, and an assessment of medication and discharge instruction compliance. Patients who received a telehealth visit were sent a brief follow-up survey at 30 days, which asked them 1) how helpful the TH visit was, 2) if they would recommend a TH follow-up to someone else, and 3) if they required additional care since their ED visit (i.e., an additional ED or urgent care visit and/or hospitalization).

At the end of the rotation, all 12 PGY-3 residents completed a short survey, administered through Survey Monkey, which solicited feedback on the educational value of the TH experience. Open-ended questions were included in the survey to capture resident comments on the benefits of TH training, ways in which the experience could be enriched, and residents' opinions on whether TH training should be included in the formal EM residency curriculum.

Data are presented as descriptive statistics. Because of its focus on quality improvement, the study was exempt from review by the Institutional Review Board (IRB) of the Sidney Kimmel Medical College of TJU.

Educational and theoretical considerations

The educational framework that informed the telehealth training program is rooted in experiential learning; furthermore, resident supervision by telehealth-trained faculty preceptors promoted reflection after telehealth clinical encounters. David Kolb’s widely quoted ‘experiential learning cycle’ informed training program design. Kolb originally represented four elements (i.e., concrete experience, observation and reflection, the formation of abstract concepts, and active experimentation) in an experiential learning circle that provides a flexible framework for instructional design [7]. The model is derived from a model of social learning that connects variability of individual learning style to flexibility in learning context [8]. In the context of the telehealth encounter, the senior resident is immersed in a concrete experience (i.e., the JeffConnect telehealth patient encounter), he/she then observes and reflects on himself/herself or others (i.e., debriefing with the faculty preceptor and/or observation of ‘modeled’ encounters by the faculty preceptor), the learner is then able to make inductive systematic conclusions or abstractions (i.e., refining frames for his/her clinical practice via the telehealth medium), which then allows the learner to empirically test the action plans that arise from the abstract concepts (i.e., applying this new practice to subsequent telehealth encounters) [8].

## Results

From August 2016 to May 2017, 624 ED follow-up visits were scheduled to be performed via telehealth. Of these, 197 patients conducted the visit (32%). Visits were conducted by all 12 PGY-3 residents. One hundred twenty-six patients (64%) were female, 98 (49.7%) patients were black. The top five chief complaints included extremity pain (11.2%), abdominal pain (8.1%), upper respiratory infection (8.1%), lacerations (7.6%), and motor vehicle accidents (7.6%) (Table 1). The average number of days between the ED visit and the telehealth follow-up call was 5.1 (interquartile range, IQR, 3-6) days. Patients reported that they were compliant with at least one part of their discharge instructions 44.7% of the time, and with medications 58.9% of the time. New prescriptions were provided in six follow-up visits (3.0%). At the end of the call, 106 encounters (54%) were “resolved,” meaning no further follow-up was necessary.

**Table 1:** Demographic and Chief Complaint Data of Patients Receiving Telehealth (TH) Follow-Up Visits After Emergency Department Discharge (N=197)

Demographic Data	N=197	%
Age	34.9 (Mean)	IQR (25-43)
Gender		
Female	126	64.0%
Male	71	36.0%
Race		
Black	98	49.7%
White	82	41.6%
Other	17	8.7%
Most Common Chief Complaints		
Extremity Pain	22	11.2%
Abdominal Pain	16	8.1%
Upper Respiratory Infection	16	8.1%
Laceration	15	7.6%
Motor Vehicle Collision	15	7.6%
Back Pain	12	6.1%
Joint Pain	11	5.6%
Chest Pain	10	5.1%
Headache	9	4.6%
Fall	8	4.1%
Cellulitis / Rash / Abscess	6	3.0%
Dizziness	6	3.0%
Gynecologic-Related Complaint	6	3.0%

A total of 104 patients were able to be reached for the 30-day follow-up (53%). On a Likert scale from 1 (low) to 10 (high), the average patient helpfulness rating was 8.2 (IQR 7.8-10), and the average patient likelihood to recommend a TH follow-up visit was 8.5 (IQR 8-10). Six patients (6%) were hospitalized within 30 days, while 23 patients (22%) had ED or urgent care visits.

Each resident completed an average of 13 TH visits (IQR 8-16.5). Ten of the 12 residents completed the post-rotation survey (response rate 83%). Seven residents (70%) agreed that there is educational value to have a TH rotation in the EM residency curriculum. Residents commented that “the experience was valuable,” that “(they) liked it better than expected,” and that it appropriately “prepared (them) for telehealth in EM,” specifically for specialized areas such as “remote and rural settings” and “assisting with EMS services.” Additional comments highlighted that the rotation “exposed them to a new medium that is in its infancy, but will likely dominate healthcare delivery in the near future.” Three residents commented that they enjoyed the patient follow-up and the ability to assess their progress post discharge.

With regards to areas for programmatic improvement in the rotation, eight residents submitted detailed descriptions on how the experience could be optimized. Open axial coding was used to organize these comments into themes (Table 2). These included integrating TH into other rotations, formalizing TH training in residency, allowing residents to get exposed with being the first provider, and maximizing the educational value of their time when the patient does not show-up for the TH encounter.

**Table 2:** Areas for Programmatic Improvement in Telehealth (TH) Training

Qualitative Themes	Specific Details
Integrate TH Training into Other Rotations	Allow resident to use TH during the EMS rotation; use TH in the urgent care setting; explore wilderness medicine applications
Create More Formalized Training	Integrate more lectures, didactics, best practices into the rotation; share more resources with residents; more detailed approach on how to incorporate TH into direct patient care
Leverage the Initial Encounter	Find ways to allow the resident to be the first provider; if possible, residents could potentially follow-up on their own patients and procedures (i.e., lacerations) through TH; have resident make decisions via TH during the undifferentiated stage of patient presentation
Maximize Down-Time	Secondary to patient cancellations, there was idle time; find ways to maximize the educational value of this down-time during the TH rotation

## Discussion

In this evaluation of a first-ever, resident-operated program for TH follow-ups of ED patient visits, we found that of the patients who completed a TH encounter, over half of the issues were resolved by the follow-up TH visit. Patients found the TH visits helpful, and would also recommend the service to others. Many patients discharged from the ED or hospital lack timely outpatient follow-up with primary care providers [9], and TH may provide a new way to provide these services, and possibly prevent repeat ED visits and hospitalizations. In addition, the TH follow-up provides patients the time to understand and review instructions and medication after their ED discharge.

As TH services become more common and accessible to patients [10-11], providers will need to have standardized training to teach how to appropriately and effectively provide these services. This is the first report of an integrated TH curriculum within an EM residency program.

Specific objectives of our program included the following:

1. Introduce resident physicians to a professional ‘tele-presence,’ including appropriate attire, technological preparation, and environmental preparation.

2. Develop the skills needed to effectively conduct TH visits (i.e., camera positioning, eye contact, and documentation).

3. Conduct appropriate histories and physical exams within the scope of TH, under the supervision and guidance of a trained faculty member.

4. Apply and critique peer-reviewed clinical pathways for TH delivery for common patient complaints.

5. Systematically troubleshoot computer and connectivity issues, and

6. Educate patients on the utility and limitations of TH.

In this novel educational program, our first class of graduating PGY-3 residents successfully met these objectives through ‘on-the-job’ training and direct supervision from EM faculty who were trained in TH delivery. Most residents agreed that TH should be incorporated into the EM curriculum, to some capacity. Comments made by residents were also insightful, and highlighted areas where improvements could be easily made to maximize the educational value of the TH experience, including better utilization of downtime.

The healthcare sector has acknowledged the increasing value added by the integrated use of TH [11-12]. Its benefits are threefold: this new means of healthcare delivery has the potential to significantly broaden access to healthcare, increase efficiencies and reduce costs, and enhance patient safety, quality of care, and ultimately, patient outcomes.

There are several limitations worth noting in our study. The sample size included in our training intervention was only limited to the PGY-3 cohort at our training site. Future plans will aim to aggregate this data with yearly data of graduating senior residents working with TH. If possible, there may be utility to compare and contrast the experience of EM resident trainees with residents from other specialties and across other institutions. The authors also wish to acknowledge that the study was possible given the successful TH program that currently exists at the host institution, to some extent, this may impact its reproducibility at other institutions. 

With regards to the overarching research question initially posed, our data supports that integrating TH training into a graduate medical education program for senior EM residents is quite feasible. In terms of program effectiveness, the Kirkpatrick Four-Level Training Evaluation Model was helpful to objectively analyze the impact of integrating TH emergency department visits into residency training. Trainees reacted well to training (Level 1: Reaction), the majority of residents enjoyed the program and agreed that it was of sound educational value. Through successive TH patient encounters, senior EM residents were able to learn how to independently facilitate a supervised TH encounter for a wide array of common patient presentations (Level 2: Learning) using specific skills to conduct the TH visit and troubleshoot connectivity issues. Senior residents were also able to apply their traditional patient care skills (i.e., extracting histories and conducting physical examinations) to the supervised TH encounter (Level 3: Behavior).

Next steps for this program will include evaluating Kirkpatrick’s Level 4 (Results) on programmatic impact. Specifically, the investigators plan to examine long-term outcomes on patients evaluated via TH (i.e., hospitalization rates, patient satisfaction ratings). Preliminary patient-specific data is encouraging: the majority of patients highly rated the TH encounter, and the majority of patients would recommend a TH visit to someone else. The investigators also plan to incorporate resident feedback into the TH experience to further improve the training program’s impact. Based on feedback provided, consideration will be made to expand the program to include first-time encounters of the undifferentiated patient via the TH platform, and appropriately link learning outcomes to ACGME milestones relevant to EM.

## Conclusions

In response to the accumulating evidence in support of new TH roles and education, we have created a feasible TH training opportunity integrated within an EM residency training that aligns with patient-centered care. In this first-year pilot program, both patients and resident providers were satisfied with this new service and new educational opportunity, respectively. Successfully optimizing this rotation has the opportunity to create a unique training opportunity for EM residents, which will help them become part of a skilled TH workforce to meet a rapidly-growing demand, and, in the process, prepare them for diverse and future-oriented job opportunities.
